# Handgrip Strength Is Associated with Specific Aspects of Vascular Function in Individuals with Metabolic Syndrome

**DOI:** 10.3390/biomedicines11092435

**Published:** 2023-08-31

**Authors:** Juan Carlos Sánchez-Delgado, Daniel D. Cohen, Paul A. Camacho-López, Javier Carreño-Robayo, Alvaro Castañeda-Hernández, Daniel García-González, Daniel Martínez-Bello, Gustavo Aroca-Martinez, Gianfranco Parati, Patricio Lopez-Jaramillo

**Affiliations:** 1 Universidad de Santander, Facultad de Ciencias Médicas y de la Salud, Bucaramanga 680003, Colombia; danielcohen1971@gmail.com (D.D.C.); javi.carreno.19@gmail.com (J.C.-R.); al.castaneda@mail.udes.edu.co (A.C.-H.); dan.martinez@mail.udes.edu.co (D.M.-B.); 2 Grupo de Investigación Ser Cultura y Movimiento, Universidad Santo Tomás-Bucaramanga, Santander 680001, Colombia; daniel.garcia@ustabuca.edu.co; 3 Department of Physical Education and Sport Sciences, Faculty of Education and Health Sciences, University of Limerick, V94T9PX Limerick, Ireland; 4 Fundación Oftalmológica de Santander, Floridablanca 811004, Colombia; paul.camacho@foscal.com.co; 5 Facultad de Ciencias de la Salud, Universidad Simón Bolívar, Barranquilla 080002, Colombia; garoca1@hotmail.com; 6 Istituto Auxologico Italuano & University of Milano-Bicocca, Department of Medicine and Surgery, Piazza Brescia, 20149 Milan, Italy

**Keywords:** handgrip, metabolic syndrome, blood pressure, isometric strength, vascular stiffness, muscle strength dynamometer

## Abstract

Background: Metabolic syndrome (MetS) is a disorder associated with an increased risk for the development of diabetes mellitus and its complications. Lower isometric handgrip strength (HGS) is associated with an increased risk of cardiometabolic diseases. However, the association between HGS and arterial stiffness parameters, which are considered the predictors of morbidity and mortality in individuals with MetS, is not well defined. Objective: To determine the association between HGS and HGS asymmetry on components of vascular function in adults with MetS. Methods: We measured handgrip strength normalized to bodyweight (HGS/kg), HGS asymmetry, body composition, blood glucose, lipid profile, blood pressure, pulse wave velocity (PWV), reflection coefficient (RC), augmentation index @75 bpm (AIx@75) and peripheral vascular resistance (PVR) in 55 adults with a diagnosis of MetS between 25 and 54 years old. Results: Mean age was 43.1 ± 7.0 years, 56.3% were females. HGS/kg was negatively correlated with AIx@75 (r = −0.440), *p* < 0.05, but these associations were not significant after adjusting for age and sex. However, when interaction effects between sex, HGS/kg and age were examined, we observed an inverse relationship between HGS/kg and AIx@75 in the older adults in the sample, whereas in the younger adults, a weak direct association was found. We also found a significant association between HGS asymmetry and PVR (beta = 30, 95% CI = 7.02; 54.2; *p* <0.012). Conclusions: Our findings suggest that in people with MetS, maintaining muscle strength may have an increasingly important role in older age in the attenuation of age-related increases in AIx@75—a marker of vascular stiffness—and that a higher HGS asymmetry could be associated with a greater vascular resistance.

## 1. Introduction

Metabolic syndrome (MetS) is associated with an increased risk of type 2 diabetes and cardiovascular diseases (CVDs). MetS is defined by the presence of at least three of the following risk factors: high waist circumference, hypertriglyceridemia, low HDL cholesterol, hypertension and dysglycemia [1]. Between 20% and 30% of the adult population can be characterized as having metabolic syndrome, which is considered a clinical picture associated with an increased incidence of arterial hypertension, atherosclerosis, left ventricular hypertrophy, diastolic dysfunction as well as an increase in premature mortality from coronary and cerebrovascular diseases [2].

While it is well established that a higher adiposity increases the risk of MetS, recent evidence also supports an association between lower muscle strength and mass, and the development of MetS and CVDs in adults [1,2,3,4] as well as a poorer metabolic risk profile in children [5]. Skeletal muscle loss and fat accumulation share a combination of factors, including increased oxidative stress, elevated inflammatory cytokines, mitochondrial dysfunction and insulin resistance (IR) [6]. It is believed that a persistent condition of these factors, particularly IR, justifies the close association between MetS and sarcopenia [7]. This can be substantiated by considering that one of the main causes of this syndrome is the increase in IR, which can be significantly exacerbated by the reduction in skeletal muscle mass—a tissue responsible for approximately 80% of glucose clearance [8]. Another reason that underscores this association is that IR is accompanied by an increase in the release of free fatty acids and the inhibition of the growth hormone (GH)–insulin-like growth factor 1 (IGF1) axis. This inhibition further hampers skeletal muscle protein synthesis. Additionally, it is believed that hyperinsulinemia caused by IR also augments the amount of myostatin, which acts to diminish skeletal muscle [8,9].

MetS is associated with an increased arterial stiffness across all age groups. This is thought to be mainly due to hormonal and metabolic abnormalities present from the onset of a state of insulin resistance—a preponderant factor that commonly accelerates vascular aging. Arterial stiffness is characterized by the loss of the elastic properties of the arteries, and while a consequence of physiological vascular aging, it can also be accelerated in a variety of pathological conditions [10,11]. Specifically, the mechanisms that promote arterial stiffness are an increase in the collagen/elastin ratio, oxidative stress, endothelial dysfunction, vascular smooth muscle proliferation, calcification, metabolic alterations, genetic mutations, epigenetic abnormalities, sympathetic activation and renin–angiotensin–aldosterone system. The increase in cardiovascular risk in patients with MetS has been associated with changes in arterial parameters, including those that determine the degree of arterial stiffness. Specifically, some studies estimate that individuals with higher arterial stiffness are estimated to have a 48% higher risk of developing cardiovascular disease [10,11,12,13].

Decreased arterial compliance and elasticity leads to an increase in arterial stiffness, a common risk factor for the development of atherosclerotic cardiovascular diseases [12,13]. The augmentation increase index (AIx) and PWV are considered the main markers of systemic arterial stiffness in the general population, and provide an estimation of vascular aging in patients with Mest. These can be measured non-invasively by tonometry, oscillometry, ultrasound, magnetic resonance imaging, with elevated values indicative of an increased arterial stiffness. The PWV is considered the “gold standard” non-invasive parameter for measuring arterial stiffness. AIx has also shown independent associations with cardiovascular events and all-cause mortality [14,15].

Some studies report an inverse relationship between muscle strength and arterial stiffness in healthy populations and in older adults, and it is suggested that endothelial dysfunction and arterial stiffness could mediate the association between muscle strength and cardiovascular events [14,15,16,17,18,19,20]. However, the association between strength and variables that relate to arterial stiffness in subjects with MetS has not been described. Furthermore, studies examining associations between strength and components of vascular function have a typically measured dynamic strength using equipment that, due either to cost and/or to time requirement for implementing the assessment, are not practical to implement in clinical settings [21]. In contrast, isometric handgrip dynamometry assessment is a relatively low cost, portable and rapid means to obtain a measure of maximal strength [22]. Handgrip strength (HGS) is the measure most commonly used in studies showing associations between low strength and the current metabolic risk profile and the risk of future CVD disease and mortality, including associations with hypertension [23,24,25,26,27]. Although HGS measures in these studies focus on the maximum HGS, interest in HGS asymmetry has increased due to evidence showing that its inclusion in analyses increases the predictive power of HGS on health outcomes such as falls, limitations functions, cognitive alterations, future chronic disease risk events and mortality [28,29,30,31,32].

Studies examining the association between HGS and arterial stiffness have found inconsistent results [20,33,34]. Furthermore, to the best of our knowledge, neither this association nor HGS asymmetry have been investigated in a MetS population. The present study aims to determine the association between maximal HGS, HGS asymmetry and aspects of vascular function in individuals with MetS.

## 2. Methodology

The present study was a cross-sectional analysis of fifty-five men and women between 25 and 54 years of age diagnosed with MetS according to the criteria of the International Diabetes Federation (three or more of the following factors: abdominal obesity (>80 cm in women/>90 cm in men), blood pressure (≥130/85 mmHg) or taking antihypertensive drugs, a fasting blood glucose between 100 and 125 mg/dL, elevated triglycerides (>150 mg/dL) and a decreased HDL (<40 mg/dL in men/<50 mg/dL in women) or taking lipid-lowering medications) [1,35]. The sample was selected through a non-probabilistic convenience sampling of the employees of two health institutions in Bucaramanga, Colombia. The participants were a subsample of subjects recruited for a randomized clinical trial of isometric strength training (EEFIT). A physiotherapist and/or nurse approached each of the potential participants in the workplace to implement the first stage of screening, which consisted of measuring the abdominal circumference with a measuring tape and blood pressure with a validated digital blood pressure monitor (Omron HEM 705 CP, **Omron** Healthcare Inc., Lake Forest, IL, US) to confirm two of the inclusion criteria before performing analyses of blood biochemistry. Those that fulfilled at least one of the additional criteria (fasting blood glucose, triglycerides or HDL) were then invited to participate, and the same professionals performed other hemodynamic and anthropometric measures and assessment of HGS. Evaluations were carried out between the months of October 2019 and January 2020.

### 2.1. Procedures

After a 15 min rest period, sitting, without crossing the legs, arterial stiffness parameters were assessed using the Mobil-O-Graph^®^ 24Hpwa device (IEM, Stolberg, Germany). The device cuff was placed on the non-dominant arm and with the arm supported at the level of the heart. The equipment initially measures blood pressure. The cuff is then inflated for approximately 10 s to the level of diastolic pressure to allow the measurement of pulse waves of the brachial artery from which several arterial stiffness parameters are indirectly estimated using the ARCSolver^®^ algorithm (Austrian Institute of Technology, Vienna, Austria). The following parameters were estimated and used in further analysis: pulse wave velocity (PWV); defined as the time difference between the start of the forward pulse wave and the beginning of the reflected wave, augmentation index at 75 bpm (AIx@75); the difference between the second and first systolic peaks, also representing the intensity of the reflection of the pulse waves, reflection coefficient (RC); the relationship between the amplitude of the reflected pulse wave and the incident pulse wave, and peripheral vascular resistance (PVR); the resistance in the circulatory system that contributes to blood pressure [36]. Height was measured using a standard height rod graduated in centimeters (cm) and millimeters (mm), to the nearest 0.1 cm. Body weight was measured using a digital scale with a precision of 100 g, and body mass index (BMI = kg/m^2^) was calculated. Fat mass (kg) and % fat was estimated using bioimpedance (BC—554 Ironman^®^, Tanita, Tokyo, Japan). Isometric HGS was evaluated using a hydraulic hand dynamometer (JAMAR^®^—5030J1, Chicago, IL, USA) with the participant seated, shoulders adducted and without rotation, with the elbow flexed at 90°, forearm and wrist in neutral position with an extension between 0 and 30° and with an ulnar deviation of 0°–15°. Three maximal voluntary contractions were performed in each hand and the highest of the three attempts was used in further analysis after normalizing for bodyweight (HGS/kg). HGS was measured alternately in the right and left hand, with a rest period of approximately one minute between measurements of the same hand [37].

We calculated an asymmetry ratio by dividing the higher HGS by the lower HGS, irrespective of hand “dominance”, to generate an absolute % magnitude value (independent of direction).

### 2.2. Statistical Analysis

Data were analyzed using Stata 12.0 (Stata Corp LCC, College Station, TX, USA). Measures of central tendency and dispersion were calculated for the quantitative variables, and absolute and relative frequencies for categorical variables. Gender differences in variables were assessed using the student’s *t*-test. We determined correlations between HGS/Kg, HGS asymmetry and arterial stiffness parameters. A multiple linear regression model was performed using three levels of interaction where HGS/Kg, age and sex were used as predictors and Aix @75, RVP, PWV, RC as response variables. An additional multiple linear analysis was performed between the arterial stiffness variables and HGS asymmetry, adjusting for gender, age, BMI, fat mass, triglycerides, glucose and HDL. An alpha level of 5% was set as significant for all analyses.

## 3. Results

The general characteristics and the metabolic profile of the study population are described in Table 1. The mean age was 43 years and 56% were women, who presented significantly lower values of grip strength (difference = −18.09 ± 4.1 Kg, *p* < 0.001), relative handgrip strength (difference = −0.06 ± 0.09 Kg/kg, *p* < 0.05), diastolic blood pressure (difference = −7 ± 10 mmHg, *p* = 0.001), systolic blood pressure (difference = −6 ± 4 mmHg, *p* < 0.001) and waist circumference (difference = −9 ± 5.4 cm, *p* = 0.001); men showed lower fat percentage (difference = −10 ± 4, *p* < 0.05), HDL (difference = −9 ± 6.1 mg/dL, *p* < 0.001) and AIx@75 (difference = −13 ± 7, *p* < 0.001).

**Figure 1:**
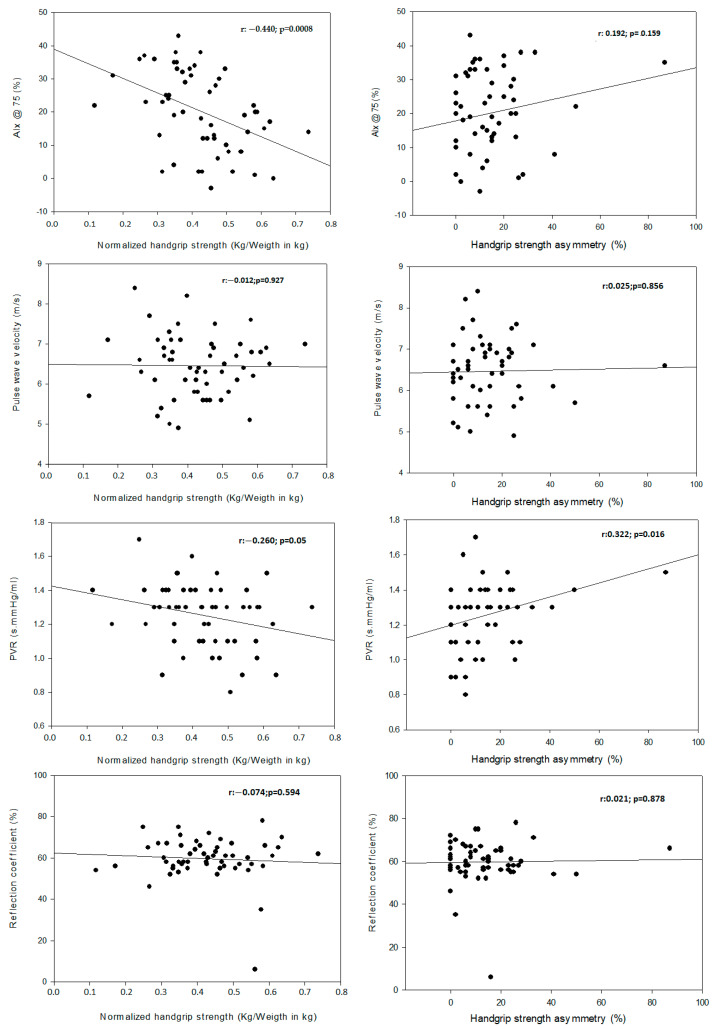
Analysis of the correlation between HGS/Kg, HGS asymmetry and indicators of arterial stiffness in individuals with MetS. Aix@75 = augmentation index at 75 bpm; PVR = peripheral vascular resistance.

Figure 1 shows negative correlations (Pearson) between HGS/kg and Aix@75 (r = −0.440, *p =* 0.0008) and PVR (r = −0.260, *p =* 0.050), and a positive correlation between HGS asymmetry and PVR (r = 0.322, *p =* 0.016). The linear regression analysis, adjusted for sex and age, and the model of covariance with three levels of interaction were not significant. The two-level interaction effect regression model showed an inverse association between HGS/kg and Aix@75 as age increased in the population (*p* < 0.05) (Table 2).

Multiple linear regression analysis adjusted for gender, age, BMI, fat mass, triglycerides, glucose and HDL showed a significant association between HGS asymmetry and PVR values (beta = 30; 95% CI = 7.02;54.2; *p* = 0.012) (Table 3).

## 4. Discussion

In the present analysis of associations between HGS and markers of vascular function in middle-aged adults with MetS, there were three principal findings. First, after adjusting for age and sex, relative strength (HGS/Kg) was not associated with any marker of vascular function. Second, we found a significant interaction effect between HGS/Kg and AIx@75 as a function of age, such that, in the older study participants (aged between 40 and 55 years old), a greater HGS/kg was inversely associated with this marker—an indicator of the state of the muscular or peripheral arteries. Third, those subjects with high HGS asymmetry were shown to be more likely to present high PVR values. This suggests that inadequate muscle strength with increasing age, as well as elevated strength asymmetry, could be predictors of arterial stiffness, at least in people with metabolic syndrome.

Women presented lower values of handgrip strength, blood pressure, lower adiposity and arterial stiffness than men. The differences evidenced in body composition, muscle strength and blood pressure between the men and women analyzed are commonly described in the literature [38]. However, the higher AIx@75 values found in the women in the present study conflict with the findings of Ogola et al. [39], who observed lower levels than in men of the same age. This may be related to the majority of women in their study being of reproductive age. Although the protective mechanisms on arterial stiffness in women of reproductive age are not fully elucidated, it is believed that hormonal factors, specifically those in relation to estrogen levels, as well as the density of their receptors, have a significant influence on vascular health. Despite this, it is important to note that the decrease in the levels of this hormone does not occur exactly at 50 years of age, which is the average age considered for the onset of menopause; therefore, the deleterious effects of estrogen decline on the cardiovascular system may begin before the natural cessation of menstruation, and could partly explain our results [40].

To our knowledge, only two studies have evaluated the association between HGS/Kg and AIx@75%, in hypertensive and diabetic subjects with a mean age of 58 and 61 years, respectively. These studies showed both lower values of grip strength and higher values of AIx@75% than those obtained in the present study, consistent with their older age and their a diagnosis with two of the chronic pathologies that are commonly associated with an increased arterial stiffness. They observed a negative correlation between HGS/Kg and AIx@75% [20,33]. Another study found a negative correlation between maximum dynamic strength and AIx@75% in individuals with mobility limitations and a mean age of 68 years [41]. This may be explained by the association between muscle function and capillary density, an important determinant of microvascular function [21,42,43].

It is also known that there is an inverse relationship between the augmentation index and the bioavailability of nitric oxide (NO), a potent vasodilator and anti-atherogenic substance produced in the vascular endothelium [44]. Aging is accompanied by a reduction in the bioavailability of NO and by an increase in the formation of peroxynitrite in smooth and skeletal muscle. The former limits blood perfusion to muscle fibers [45,46] while the latter decreases contractile force and increases susceptibility to muscular fatigue, [43] providing potential explanations for the observed associations between muscle function and AIx@75% as age increases in people with Mets. Alternatively, microvascular blood flow is a determinant of anabolic processes, and as such, a diminished vascular function might accelerate the ageing-related losses of muscle mass, and as a consequence, a reduction in muscle strength and function. In addition, microvascular dysfunction can alter metabolism, mainly developing insulin resistance, a factor that affects arterial stiffness and accelerates arterial aging [18,47,48,49].

Another of the possible mechanisms that explain the relationship between HGS and markers of stiffness observed relates to the understanding of muscle as an endocrine organ, having a better state and which can promote the production of myokines (such as interleukin-15, myostatin and irisin), which improve the processes of the regulation of fats and carbohydrates, thus favoring endothelial function and neovascularization, which in turn is accompanied by lower values of arterial stiffness [50]. In addition, arterial stiffness, as well as the attenuation of NO bioavailability that commonly accompanies it, could reduce blood flow to resting muscle tissue and hyperemia due to physical exercise by approximately 45%, which leads to a reduced supply of oxygen and nutrients to the muscle, thus limiting its contractile ability [41,51] These conditions have also been shown to affect satellite cell activation and therefore the hypertrophy of skeletal muscle fibers [52]. Additionally, the literature reports that a decrease in NO synthesis may negatively affect muscle excitation–contraction coupling, by reducing the activity of dihydropyridine receptors located in the T-tubules of the cytoplasmic membrane, as well as the activity of the enzyme calcium—ATPase—and the release of calcium from the sarcoplasmic reticulum through an alteration in ryanodine receptors [53,54,55].

The average asymmetry of HGS in the studied population was 14%, showing above the 10% threshold considered to be significant asymmetry by a number of studies [28,29,30,31,32]. Based on this cut-point, 51% of those evaluated presented an elevated asymmetry, similar to that reported previously in samples over 50 years of age [28,56]. However, it is important to highlight that there is no unified diagnostic criterion for asymmetry, considering the limited epidemiological information on this topic. The association between HGS asymmetry and PVR may be related to the finding that increased asymmetry has been shown to be a marker of decreased muscle function [28,57], a condition that may be accompanied by endothelial and smooth muscle vascular deterioration, mainly at the arteriolar level where peripheral vascular resistance is regulated [42,43,58]. In addition, individuals with a reduced muscle function are likely to also present with a lower capillary density [59], a factor that could also promote an increase in PVR [60]. While potential mechanisms linking strength asymmetry and cardiovascular disease risk are not clear, several studies have shown that a higher HGS asymmetry is associated with poorer cardiovascular health outcomes [61]. Our findings align with this and supports the need for the inclusion of asymmetry measures in the further investigation of potential associations between strength and cardiovascular health in those with, and without MetS.

The methodology and results of the present study do not permit us to determine the physiological and/or molecular mechanisms that describe the influence of HGS on arterial stiffness. However, there are common factors that may contribute to low muscle strength, as well as an increased arterial stiffness, including oxidative stress, insulin resistance, increased body fat percentage, endothelial dysfunction and the presence of higher levels of circulating inflammatory markers (elevated levels of C-reactive protein, interleukin-6, D-dimer, factor VIII) [33,62,63,64].

Amongst the other results that emerged in the interaction analysis, there was a direct relationship between HGS/kg and AIx@75% in people aged 25 to 40 years, an association that was stronger in women. This finding is contrary to the prior literature, showing that regardless of age, higher strength is related to a better vascular status [21,65,66,67]. Associations between grip strength and PWV are reported primarily in subjects older than 50 years, a potential explanation for the lack of association in the present study [14,18,48,65,66,67,68,69,70]. In addition, as this marker is more representative of lower-body arterial stiffness, it may be more associated with measures of lower body strength than with HGS, which correlates more strongly with upper extremity strength [71,72].

Studies that reported associations similar to that of the present investigation used diverse evaluation modalities. Most evaluated vascular health status using tonometry and plethysmography [21,41,65,66,67], while one used oscillometry [20], which is the modality employed in the present study and which is shown to have good psychometric properties compared to invasive and non-invasive evaluation methods [73,74]. Despite the predictive value shown by arterial stiffness and grip strength on cardiovascular health status, as well as the adequate psychometric properties of the different tools used to assess these markers, their use is mainly described in the research context, with little applicability in clinical practice. There is therefore a need to determine if the data obtained with these tools can facilitate clinical decisions, considering that they are economical and versatile. In addition, they have shown to be useful for the early identification of biological aging before cardiovascular disease is evident, making their use as a screening/preventive tool attractive—HGS in particular, as it is highly reproducible and is faster and less prone to errors. Therefore, further research examining the association between the two variables of interest analyzed in this study might further develop our understanding of the relationship between HGS and CVD in subjects with MetS. Furthermore, it is conceivable that targeted interventions and/or advice to improve muscle strength in those diagnosed with low HGS may contribute to an improved arterial health [18,41,75,76,77]. Particularly promising are the results of low-intensity isometric exercise in reducing systolic blood pressure [78,79], with some evidence that improvements in specific aspects of vascular function contribute to this effect [80].

## 5. Study Limitations

The study’s limitations include the small sample size, which may have limited our ability to confirm other associations, the lack of control over the use of antihypertensive drugs or hormone replacement therapies—a potential confounding influence on the associations observed—and finally, the cross-sectional design, which prevents us from establishing causality. However, recognizing that HGS, HGS asymmetry and arterial stiffness are markers of biological age, it is possible that there is not a unilateral or bilateral causal relationship between these variables, and the possible association observed is due to them measuring the same characteristic.

## 6. Conclusions

Our findings suggest that in people with MetS with increasing age, a lower relative handgrip strength is increasingly associated with higher AIx@75; moreover, HGS asymmetry could also be a marker of arterial stiffness. The causality and direction of causality remain to be determined. In this population, at an elevated risk of diabetes and CVDs, further studies are needed to examine whether isometric exercise interventions that promote the development of strength or reduce strength asymmetry can improve AIx and/or other markers of vascular function/stiffness and whether these changes may have an impact on the outcome. In addition, the positive interaction between HGS/kg and Aix observed in the younger participants warrants further investigation. Finally, further work is needed to understand causality and the pathophysiological mechanisms involved.

## Figures and Tables

**Table 1:** Characteristics of the study population.

Variable	Female (n = 31) Mean (SD)	Male (n = 24) Mean (SD)	Total (n = 55) Mean (SD)
Age (years) %	44.48 (6.92)	41.42 (7.02)	43.15 (7.07)
BMI (kg/m^2^)	29.25 (4.93)	29.10 (2.80)	29.18 (4.10)
Waist circumference (cm)	89.27 (9.02)	98.52 (6.69) *	93.31 (9.26)
Fat mass (%)	37.13 (6.44)	27.36 (3.98) *	32.86 (7.33)
Handgrip strength (kg)	25.74 (6.17)	43.83 (6.68) *	33.64 (11.05)
Relative handgrip strength (kg/weight in kg)	0.36 (0.10)	0.52 (0.09) *	0.43 (0.12)
Handgrip strength asymmetry (%)	15.8 (17.1)	11.8 (11.4)	14.0 (14.9)
Glucose (mg/dL)	99.25 (10.37)	101.70 (10.45)	100.32 (10.38)
TC (mg/dL)	196.31 (37.69)	190.97 (36.62)	193.98 (36.98)
LDL (mg/dL)	131.13 (33.42)	123.88 (28.35)	127.97 (31.24)
HDL (mg/dL)	44.69 (10.37)	36.48 (7.29) *	41.11 (9.97)
Triglycerides (mg/dL)	183.56 (73.39)	244.07 (174.98)	209.97 (130.20)
Systolic blood pressure (mm/Hg)	113.61 (7.89)	121.58 (10.12) *	117.09 (9.70)
Diastolic blood pressure (mm/Hg)	72.74 (6.93)	78.96 (6.52) *	75.45 (7.38)
RHR (bpm)	74.23 (7.93)	72.25 (7.16)	73.36 (7.60)
PVR (s.mmHg/mL)	1.28 (0.20)	1.22 (0.17)	1.25 (0.19)
Reflection coefficient (%)	61.23 (6.94)	57.62 (13.57)	59.65 (10.42)
Aix@75 (%)	25.65 (11.17)	12.96 (9.22) *	20.11 (12.08)
Pulse wave velocity (m/s)	6.50 (0.87)	6.41 (0.62)	6.46 (0.77)

SD: Standard deviation; PVR: peripheral vascular resistance; Aix@75: augmentation index at 75 bpm; BMI: body mass index; LDL: low-density lipoprotein; HDL: high-density lipoprotein; RHR: resting heart rate; TC: total cholesterol; * *p* < 0.05, *t*-test.

**Table 2:** Univariate and multivariate linear regression coefficients of HGS/kg, age and sex with indicators of arterial stiffness.

Response	Characteristic	Univariate	Multivariate
		ẞ (CI 95%), *p* value	ẞ (CI 95%), *p* Value
Aix@75	Male	−12.69 (−18.34 to −7.03), *p* < 0.001 *	−73.71 (−127.11 to −20.31), *p* = 0.008 *
	Age	0.16 (−0.31 to 0.63), *p* = 0.488	2.42 (0.61 to 4.23), *p* = 0.010 *
	HGS	−43.96 (−68.66 to −19.25), *p* = 0.001 *	314.56 (76.48 to 552.63), *p* = 0.011 *
	I-HGS_Age	−0.73 (−1.24 to −0.22), *p* = 0.006 *	−7.40 (−12.51 to −2.28), *p* = 0.005 *
	I-HGS_Sex	−23.66 (−34.43 to −12.88), *p* < 0.001 *	3.24 (−59.33 to 65.80), *p* = 0.918
	I-Sex_Age	−0.29 (−0.43 to −0.16), *p* < 0.001 *	1.42 (0.21 to 2.62), *p* = 0.022 *
PVR	Male	−0.07 (−0.17 to 0.03), *p* = 0.187	−0.93 (−1.92 to 0.05), *p* = 0.064
	Age	0.00 (−0.00 to 0.01), *p* = 0.516	0.03 (−0.01 to 0.06), *p* = 0.125
	HGS	−0.40 (−0.81 to 0.01), *p* = 0.055	2.90 (−1.50 to 7.30), *p* = 0.192
	I-HGS _Age	−0.01 (−0.01 to 0.00), *p* = 0.135	−0.08 (−0.17 to 0.02), *p* = 0.108
	I-HGS_Sex	−0.12 (−0.31 to 0.07), *p* = 0.206	0.44 (−0.72 to 1.60), *p* = 0.449
	I-Sex_Age	−0.00 (−0.00 to 0.00), *p* = 0.243	0.02 (−0.01 to 0.04), *p* = 0.138

β = Coefficient for linear regression; HGS: body weight-normalized handgrip strength; Aix@75 = augmentation index at 75 bpm; PVR: peripheral vascular resistance. I-HGS_Age: Interaction between HGS and age adjusted for sex; I-HGS_Sex: interaction between HGS and sex; I-Sex_Age: interaction between sex and age. * *p* < 0.05.

**Table 3:** Univariate and multivariate linear regression coefficients of handgrip asymmetry with indicators of arterial stiffness.

Variable	Crude Model	Fully Adjusted Models ^†^
	ẞ (CI 95%), *p*-Value	OR (95%CI), *p*-Value
Aix@75 (%)	0.23 (−0.09; 0.57), 0.159	0.25 (−0.16; 0.68), 0.227
PWV (m/s)	0.48 (−4.87; 5.84), 0.856	10 (−0.94; 21.5), 0.072
RC (%)	0.03 (−0.36; 0.42), 0.878	0.04 (0.39; 0.48), 0.824
PVR (s.mmHg/mL)	26 (4.94; 46.7), 0.016 *	30 (7.02; 54.2), 0.012 *

CI: Confidence interval; Aix@75 = augmentation index at 75 bpm; PVR: peripheral vascular resistance; † fully adjusted models controlled for age, sex, BMI, fat mass, triglycerides, glucose; HDL: high-density lipoprotein. * *p* < 0.05.

## Data Availability

Data are available upon request due to privacy/ethical restrictions.
